# Slope Micrometeorological Analysis and Prediction Based on an ARIMA Model and Data-Fitting System

**DOI:** 10.3390/s22031214

**Published:** 2022-02-05

**Authors:** Dunwen Liu, Haofei Chen, Yu Tang, Chao Liu, Min Cao, Chun Gong, Shulin Jiang

**Affiliations:** School of Resources and Safety Engineering, Central South University, Changsha 410000, China; dunwen@csu.edu.cn (D.L.); 205512072@csu.edu.cn (H.C.); 195512139@csu.edu.cn (C.L.); 205512136@csu.edu.cn (M.C.); gongchun@csu.edu.cn (C.G.); 195511019@csu.edu.cn (S.J.)

**Keywords:** micrometeorological monitoring, ARIMA model prediction, slope, data-fitting system, atmospheric meteorological

## Abstract

The rapid development of highway engineering has made slope stability an important issue in infrastructure construction. To meet the needs of green vegetation growth, ecological recovery, landscape beautification and the economy, long-term monitoring research on high-slope micrometeorology has important practical significance. Because of that, we designed and created a new slope micrometeorological monitoring and predicting system (SMMPS). We innovatively upgraded the cloud platform system, by adding an ARIMA prediction system and data-fitting system. From regularly sensor-monitored slope micrometeorological factors (soil temperature and humidity, slope temperature and humidity, and slope rainfall), a data-fitting system was used to fit atmospheric data with slope micrometeorological data, the trend of which ARIMA predicted. The slope was protected in time to prevent severe weather damage to the slope vegetation on a large scale. The SMMPS, which upgrades its cloud platform, significantly reduces the cost of long-term monitoring, protects slope stability, and improves the safety of rail and road projects.

## 1. Introduction

An important issue in infrastructure construction is slope stability [1,2,3], which involves ecological restoration, economy and landscaping, and slope protection with vegetation technology [4,5,6], usually building green slopes by planting trees and grass between soil beams and frames. Slope micrometeorology determines the growth trend of the protection vegetation, which affects the stability of the slope [7].

The national weather station can provide regional meteorological data, but micrometeorology is affected by many factors [8], so standardized global weather station data cannot accurately describe the slope micrometeorological data. Tall trees and vegetation on the slope can block sunlight and significantly reduce the temperature and humidity of shaded areas. Extreme weather and an abrupt change in micrometeorology have an important influence on the growth of slope vegetation [9,10], resulting in its destruction and a large number of exposed soil and rock slopes. Soil erosion causes an ecological imbalance, which greatly raises the requirements of slope protection design and ecological protection [11,12,13,14]. Therefore, the long-term monitoring of slope micrometeorology is important for maintaining slope stability.

Climate monitoring produces a lot of data about climate change, and meteorological trends. Talakh [15] used climate monitoring information technology, which combined satellite observation methods with climate station observations, to form an array of input data by considering their spatio-temporal characteristics, specifically the location, altitude, and type of the underlying surface. Based on remote-sensing space technology, Liu [16] combined airborne remote sensing and ground observation to conduct real-time monitoring and climate prediction to prepare for extreme weather events in China’s “Belt and Road” region. This type of climate monitoring combines weather stations with some new science and technology that can effectively monitor climate in real time, but it is expensive, technically complicated and only available to countries and large enterprises. Wild [17] developed a new temperature and humidity recorder that simulated small herbaceous plants, from which data were collected every 15 min and stored for 15 years. Holden [18] used a low-emission radiation shield to measure temperatures in harsh outdoor environments and, in contrast to a nearby remote automatic weather station, considered this low-cost climate monitoring suitable for the outdoor monitoring of surface air temperatures. The cost of this type of climate monitoring instrument is low and is suitable for small enterprises and individuals to monitor the climate, but the operation is complicated. The data need to be read and processed manually, which greatly increases the difficulty of the work [19,20], as. accuracy and actual situations have certain deviation. Therefore, how to reduce the cost and difficulty of slope micrometeorological monitoring has become an urgent problem to be solved at the present stage.

Slope micrometeorological monitoring includes a series of data such as air temperature, humidity, wind speed and soil temperature and humidity [21,22,23]. Monitoring sensors and hardware are used to provide valuable information about the behavior of slope plants and their requirements in real time [24]. For this, wireless sensors do not require cable connections, and this can effectively reduce costs. Through the flexible deployment of wireless monitoring sensor equipment, it is possible to monitor challenging environments such as high-tree crowns and high slopes for better data monitoring. Therefore, the wireless sensor is considered to be one of the most effective instruments for slope micrometeorological monitoring [25,26,27].

In this article, we innovated and upgraded the cloud platform system of wireless sensors. We built an ARIMA time-series prediction system and data-fitting system onto the sensor cloud platform. The data-fitting system can fit and process meteorological and slope-micrometeorological data to obtain a comparison between them. The ARIMA prediction system can realize high-precision predictions of partial slope micrometeorological data, reducing the long-term sensor monitoring time. Compared with traditional climate monitoring sensors, the SMMPS system monitors and records slope micrometeorology data in real time and better realizes unmanned automatic monitoring and data analysis, thereby greatly reducing labor and monitoring costs.

## 2. Structure of SMMPS

**Figure 1:**
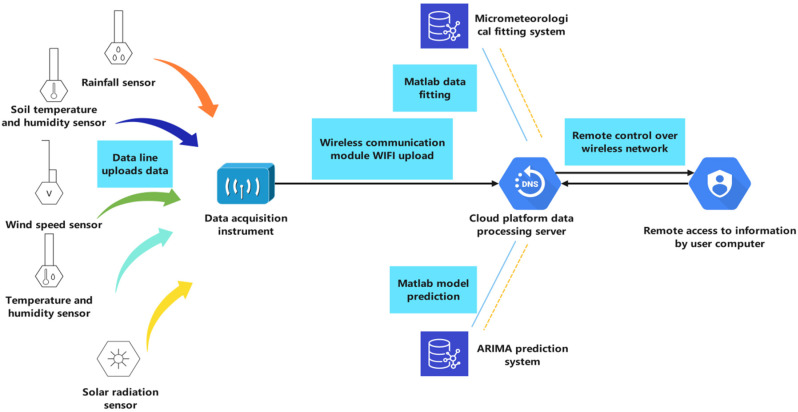
Operation process and overview of slope micrometeorological sensor-processing system.

In this part, we describe the structure of the slope micrometeorological sensor processing system in detail. Figure 1 provides the operation process of the SMMPS. It explains the working principle and operation mode of the slope micrometeorological monitoring module, and introduces the micrometeorological data-fitting and ARIMA prediction system of the cloud platform server.

### 2.1. Slope Micrometeorological Monitoring Module

The slope micrometeorological monitoring module includes a bracket, acquisition device, monitoring sensors and solar-power supply system. To maintain the stability and safety of the system, we chose a portable and stable tripod bracket. We fixed the bottom of the fixing bracket to the top of the slope without shelter manually using expansion screws. The top of the fixed bracket was equipped with a horizontal vacuole indicator, through which the bracket was levelled to ensure more accurate monitoring results.

The environmental monitoring system comprises the following sensors: rainfall, soil temperature and humidity, wind speed, atmospheric temperature and humidity, and solar radiation. All sensors are GPRS/4G models manufactured by Prysons. The rainfall sensor model is the pulse type; the diameter of the rain gauge tube is Φ 200 mm; the rain mouth is made of ABS engineering plastic; and the lag water produces small errors. The soil temperature measurement range is from −40 to about 80 °C; the declaration accuracy is 0.5 °C, and the protection class is IP68. The measurement range of the wind speed sensor is 0–70 m/s, and the accuracy is ± 0.2 m/s. The temperature range of the atmospheric temperature sensor is from −40 to about 60 °C; the declaration accuracy is 0.5 °C; and the protection class is IP65. The air humidity sensor ranges from 0 to 100% RH, and the declaration accuracy is ±2% RH. Solar radiation sensor adopts high-precision photosensitive element, wide spectrum absorption, measuring range of 0–1800 W/m^2^.

Real-time environmental data are collected through multiple sensors in the monitoring system. Some sensors collect data for upload to the acquisition instrument through the data line. Considering the cost saving and data transmission difficulty, other sensors use a 4G LTE module to upload. Compared with the 3G module, LTE takes the OFDMA as the core, reduces the delay in data uploading, eliminates the wireless network controller, and adopts a flat network architecture. This structure can make data transmission more stable and reduce the transmission failure rate. The data uploaded by the sensor will be sent to the cloud platform server by a wireless communication module at a regular time every day. The staff can view and download the test data remotely by accessing the cloud platform server on the client computer. The ARIMA system and micrometeorological fitting system of cloud platform server are also used to fit data and forecast trends to obtain the long-term monitoring and prediction of slope vegetation growth environment.

In addition, to ensure that the wireless communication system in the acquisition device uploads data to the cloud server stably and regularly, the whole monitoring module is equipped with a solar-powered supply system.

### 2.2. Cloud Platform Server

The cloud platform server is composed of s micrometeorological analysis system, ARIMA prediction system and data uploading and storage system.

#### 2.2.1. Micrometeorological Analysis System

The micrometeorological analysis system automatically processes the slope-monitoring data and the data downloaded from the China Meteorological Data Service Centre (CMDSC), an authoritative and unified shared-service platform for China’s Meteorological Administration to open up its meteorological data resources to domestic and global users.

The analysis system includes a library of fitting models, including linear-regression Fourier-series models. The correlation between micrometeorology and meteorology can be analyzed, and then the slope micrometeorology can be predicted according to the CMDSC data, and the relationship between meteorological and micrometeorological data can be established. Finally, the monitoring meteorological data of the CMDSC are input into the fitting formula to obtain the slope micrometeorological data. In this way, the field monitoring time and cost is greatly reduced.

The time series of meteorological data has obvious short-term fluctuations with a period of one day, so temperature data are processed by a central moving average. If the sampling frequency of the series is k hours per time, a total of n (*n* = 24/k) times are collected at each measuring point every day for n number of periods.

After processing the original data with the central moving average formula, a smooth time series of meteorological and micrometeorological parameters can be obtained, after which the parameter database is established on the cloud platform server. Finally, the data in the database are imported into the micrometeorological analysis system for fitting, and the trend term model and formula can be obtained.

#### 2.2.2. ARIMA Prediction System

The Autoregressive Integrated Moving Average (ARIMA) model, first proposed and described by Box Jenkins in 1976, is a time-series prediction method that can be applied to small samples [28]. It is constructed by obtaining a stationary time series through differences, including an autoregression (AR) model, moving average (MA) model and a difference method (I). ARIMA showed the advantages of strong robustness, short time-series predictability, simplicity and practicality, and is widely used in the prediction of various disciplines [29,30,31]. ARIMA has three features: p, d and q, where p is the order of the AR term; q is the order of the MA term; and d is the difference-order model required to make time series stable. To establish an ARIMA model, the equations of AR and MA should be determined according to the values of p, d and q. We input meteorological data collected from CMDSC into Matlab to establish the appropriate ARIMA model and predicted trends for temperature, humidity and other data. These were combined with the micrometeorological analysis system, and the trend of each slope in the micrometeorological database was obtained. Since the test site was located in the eastern coastal area, the vegetation planted should be able to resist strong typhoons and withstand short-term strong solar radiation as well as high and low temperature effects. According to the ARIMA prediction results, it accurately gave 5–10 days advanced warning of severe weather such as high-temperatures above 35 °C, low temperatures below 5 °C, strong solar radiation above 35 MJ/m^2^ and typhoons with a wind speed greater than 8 m/s at the slope.

**Figure 2:**
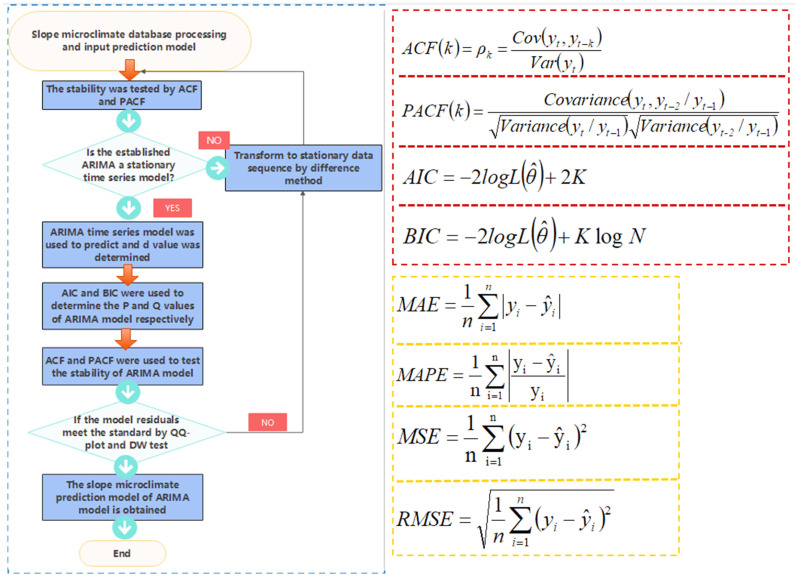
Slope micrometeorological ARIMA model flow chart and judgment formula. Where the lag k refers to the correlation between the observed data with an interval of k time periods. $logL\left(\hat{\theta }\right)$ is the likelihood function; K is the total number of model parameters; N is the number of observations; $\hat{y_{i}}$ is the model’s predicted value; $y_{i}$ is the actual value. The established regression model was evaluated according to the mean absolute error (MAE), mean squared error (MSE) and root mean square error (RMSE).

Figure 2 is the operation flow chart of the ARIMA model. According to the specific flow, a relatively stable ARIMA time-series prediction model of temperature, humidity and micrometeorological characteristics can be obtained.

The premise of establishing a time-series ARIMA model was to maintain the stationarity of data so that it could be tested by the autocorrelation function (ACF) and partial autocorrelation function (PACF). The ACF reflects the correlation between time series data of two different moments, which are affected by a variety of random variables, whereas the PACF excludes the influence of other random variables and simply measures the correlation between current time-series data and the hysteresis value.

After time-series stability is determined, the ARIMA model needs to be established, and the p and q values ordered. The purpose of model selection is to strike a balance between the model’s complexity its ability to describe the data set. The Akakpool information criterion (AIC) and Bayesian information criterion (BIC) are the most commonly used methods for determining p and q values of hierarchical models. They are composed of a model sample number and penalty term. When training the model, an increase in the number of samples increases the complexity of the model; however, if the model becomes too complex, overfitting can result, so the penalty term is added to limit over-complexity. Of the two criteria, the BIC added a larger penalty when the amount of data was large. Therefore, a better model can be selected by combining the AIC and BIC.

The Quantile–Quantile Plot (QQ-Plot) test and Dubin–Watson test were used to carry out a residual test on the model. QQ-plot added a normal distribution test line to determine if the sample data fell near the normal distribution line. If it deviated too much from the straight line, the model was unreasonable. The Dubin–Watson test is a test statistic to diagnose whether the residual ARIMA model had autocorrelation.

Finally, the prediction accuracy of the ARIMA model was evaluated by the mean absolute error (MAE), mean absolute percentage error (MAPE), mean squared error (MSE) and root mean square error (RMSE).

## 3. Experimental Verification

To validate the accuracy of the SMMPS, we need to apply it to a slope-engineering project that required long-term micrometeorological slope monitoring. To stabilize the slope, beautify the environment, and optimize the vegetation scheme with good environmental adaptability, a slope vegetation protection and micrometeorological monitoring system was applied.

### 3.1. Project Summary

We established a micrometeorological monitoring system on a high slope (29°8′ N, 120°49′ E) with an elevation difference of 8.2 m and a slope angle of 58.4°. The experimental area belongs to the subtropical monsoon climate zone, which has high temperature and rain in summer and mild and little rain in winter. It also has good biodiversity: forest, ocean and wetland ecological systems. The forest is rich in vegetation, and the tall vegetation has a great influence on the temperature and humidity and solar radiation monitoring; however, typhoons and rainstorms disturb its stability. Therefore, a project with significantly different slope micrometeorology and meteorology was selected as the experimental object to research the practical effect of the SMMPS on engineering.

### 3.2. System Layout

**Figure 3:**
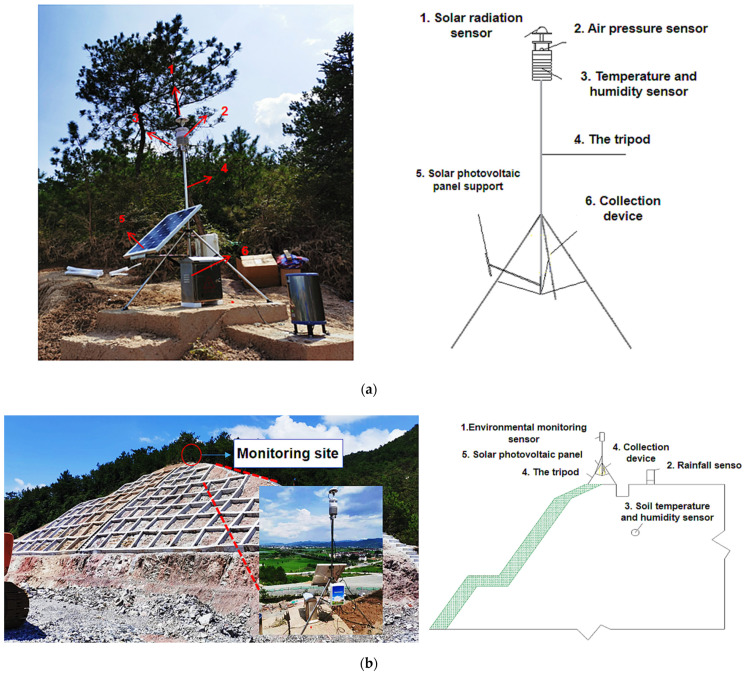
The sensor node in the experimental setup consists of multiple sensors, fixed brackets, wireless communication system and solar energy supply system. The facility is located at Hangzhou Meteorological Station 58,457 (30°14′ N, 120°10′ E). (**a**) Slope micrometeorological environment monitoring system instrument and local schematic diagram. (**b**) Slope micrometeorological environment monitoring point layout.

Figure 3 is a partial diagram of the environmental monitoring system. The environmental temperature and humidity sensor, wind-speed and direction sensor, and solar radiation sensor in the slope micrometeorological monitoring module were installed on the top of the slope using a tripod bracket. The surrounding trees affected the monitoring of solar radiation, wind speed, rainfall and other factors. The atmospheric temperature sensor was placed at the top, and to accurately monitor soil salinity, temperature and humidity, a sensor was put inside a 50 mm diameter, 2 m deep hole and covered with soil. The rainfall sensor was placed at the top of the slope without shelter. The solar-power supply system consisted of photovoltaic panels, controllers, battery banks, debuggers and DC/AC inverters. The photovoltaic panels were tilted to the south to maximize energy capture. A controller protected the charge and discharge of the colloidal battery, which was placed in a stainless steel box and fixed to the ground. Because it is maintenance-free and has lower environmental effects, this battery was suitable for the solar-power supply system in the unattended slope area.

### 3.3. Micrometeorological Monitoring Data

**Figure 4:**
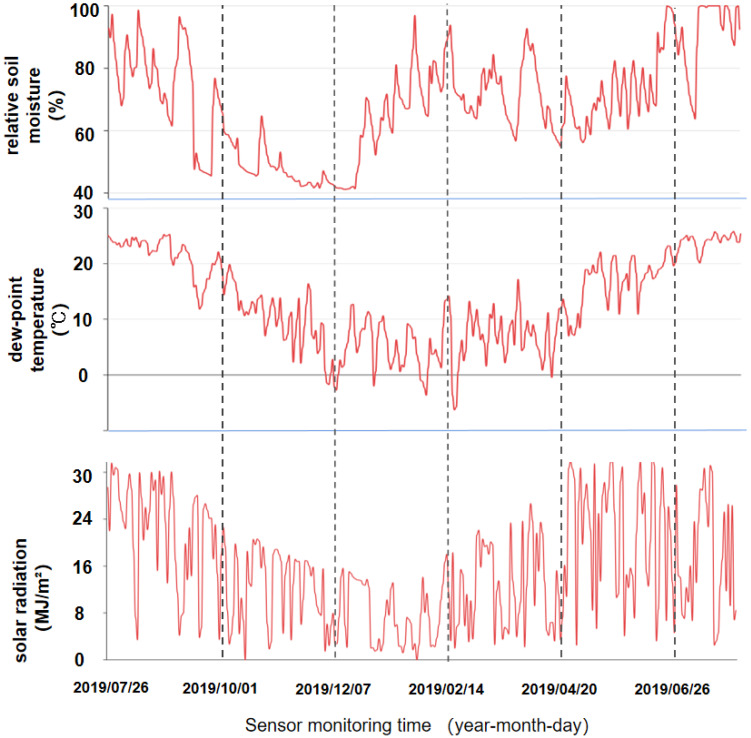
The slope was monitored for one year with meteorological data that could not be found in the Hangzhou station atmospheric data. Each image represents a single meteorological unit of monitoring data. From top to bottom are the relative soil moisture, dew point temperature and solar radiation intensity.

**Figure 5:**
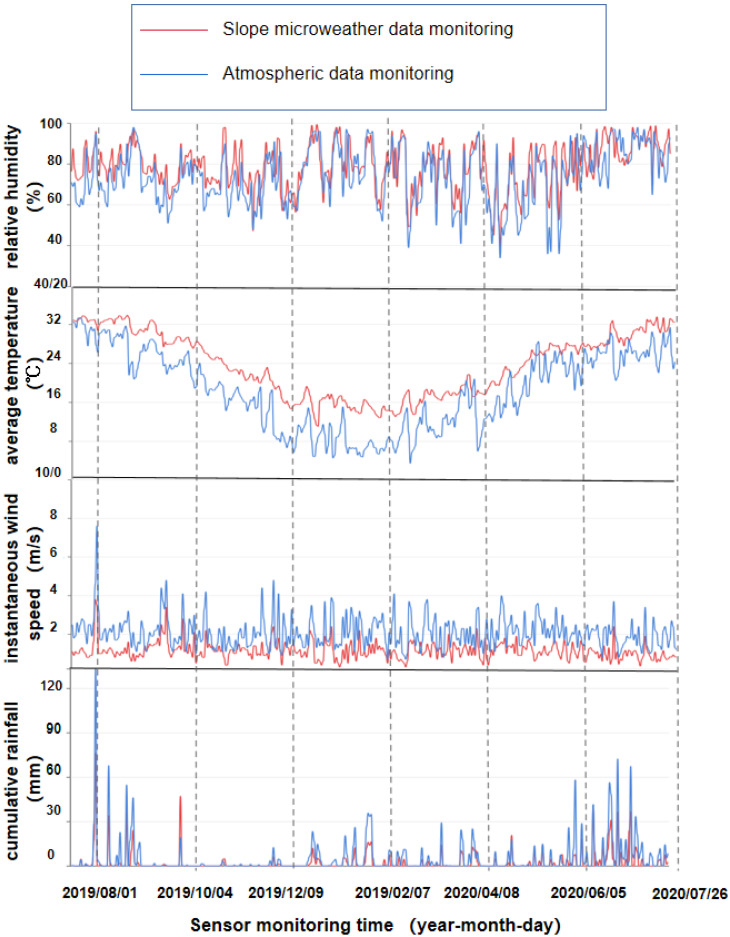
Atmospheric data from the Hangzhou station were collected for one year and the image was contrasted with the slope micrometeorological data. Each image represents a single meteorological unit of monitoring data. From top to bottom are relative atmospheric humidity, mean atmospheric temperature, instantaneous wind speed, and cumulative rainfall.

We began to monitor the slope micrometeorological data on 26 July 2019 and continued until 30 August 2020. Monitoring was done every half an hour every day for the long-term monitoring of the environmental temperature, humidity, wind speed and direction, rainfall, solar radiation, soil temperature and humidity, and salinity of the slope project. We plotted an average value of 48 pieces of monitoring data a day into a data graph. Figure 4 shows that the sensors collected data on soil moisture, dew point temperature, and solar radiation. To analyze the data better, the atmospheric meteorological data of Hangzhou Station 58,457 (30°14′ N, 120°10′ E) from July 2019 to August 2020 were collected from the CMDSC. The atmospheric meteorological data was monitored every 3 h, and the atmospheric data was compared with the slope micrometeorological monitoring data. Figure 5 shows a comparison of data from the slope micrometeorological sensor with atmospheric meteorological data, including relative humidity, mean temperature, accumulated rainfall, and instantaneous wind speed.

### 3.4. Micrometeorological and Atmospheric Data Fitting

**Figure 6:**
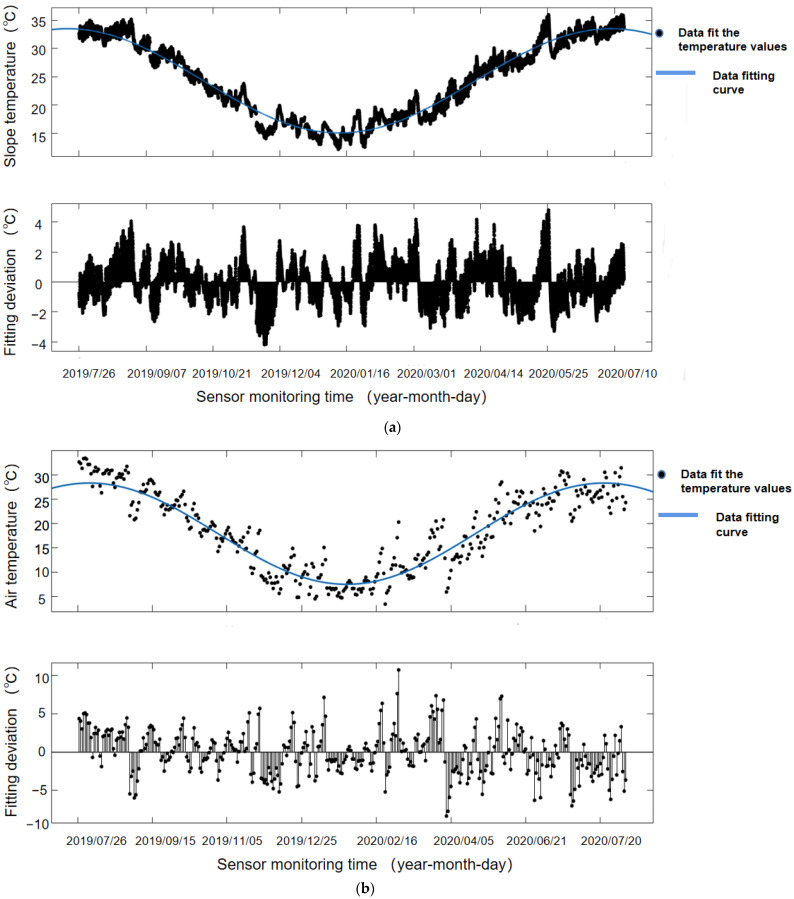
The data-fitting system of cloud platform server is used to fit the slope micrometeorological temperature monitoring data and atmospheric temperature data, in which the blue curve represents the Fourier function fitting curve, and the black represents each data point. (**a**) Fitting curve and fitting deviation of slope micrometeorological temperature monitoring data; (**b**) Fitting curve and fitting deviation of atmospheric temperature monitoring data.

Figure 6 shows the fitting image of slope micrometeorological and meteorological data. The smoothed temperature series as a whole showed a change rule of a quasi-trigonometric function. After various fitting results, the Fourier series was found to be the best fit. The Fourier series expansion is expressed as

$$T_{t}=a_{0}+\overset{\infty }{\underset{n=1}{\sum }}\left[a_{n}\cos\left(n\omega t\right)+b_{n}\sin\left(n\omega t\right)\right]$$

where $T_{t}$ is the temperature obtained after smoothing °C; t is serial number of the data point, with 00:00 on 26 July 2019 as the first serial number (*t* = 1) and 00:30 on 26 July as the second (*t* = 2) and so on; $a_{0},a_{n},\;b_{n},\;\omega$ was the fitting parameter.

The micrometeorological slope temperature and air temperature data were put through Matlab2019 software after the smooth sequence of curve fitting. In Table 1, the meteorological slope data in first-order Fourier goodness-of-fit (R^2^) was above 0.95, and the air temperature data was above 85%. Therefore, the first-order Fourier was used for fitting; that is, *n* = 1. The circular frequency ω of the micrometeorological temperature series was about 0.0003868, and the circular frequency ω of the atmospheric temperature data series was about 0.01823, and the goodness of fit R^2^ was better.

$$T_{t}=a+b\cdot \cos\left(\omega \cdot t\right)+c\cdot \sin\left(\omega \cdot t\right)$$

$$b\cdot \cos\left(\omega \cdot t\right)+c\cdot \sin\left(\omega \cdot t\right)=\sqrt{b^{2}+c^{2}}\cdot \sin\left(\omega t+\varphi \right)$$

$$T_{t}\left(t\right)=T_{m}-\Delta T_{R}\sin\left(\omega t+\varphi \right)\;$$

where, *a*, *b* and *c* are fitting parameters; and $T_{m}=a$; $\Delta T_{R}=\sqrt{b^{2}+c^{2}}$; $\varphi =\tan^{-1}\left(b/c\right)$.

The above formula is the trend-fitting function of the temperature time series. According to the above formula, the smoothed sequence fitting function is

$$T_{t}(slope\;microclimate)=24.27-9.246\sin\left(0.0003868\;t-82.39\right){\;R}^{2}=0.9549$$

$$T_{t}(air\;data)=17.93-10.367\cos\left(0.01823\;t-81.75\right){\;R}^{2}=0.8681$$

The relationship between micrometeorological slope trend-fitting function and atmospheric data-fitting function was constructed.

$$T_{t}(sm)=24.27-9.246\sin\left(0.0003868\;\frac{\cos^{-1}\left(\frac{Tt\left(ad\right)-17.93}{\left(-10.367\right)}\right)+81.75}{0.01823}-82.39\right)$$

Due to the disordered fluctuation of hourly variation values of the other atmospheric meteorological data, the relationship between mathematical functions and meteorology could not be effectively fitted. Therefore, a linear relationship between meteorological and micrometeorological data was established, and their linear models are discussed. The research method was the unitary linear regression model

Y=a+bx

where, *a* and *b* are unknown fitting parameters. Set $\left(T_{a,1},T_{\mu ,1}\right),\;\;\left(T_{a,2},T_{\mu ,2}\right),\;\;\cdots ,\;\;\left(T_{a,n},T_{\mu ,n}\right)$ as samples.

$$T_{\mu ,i}=a+bT_{a,i\;}\;i=1,2,\cdots n$$

Micrometeorological data and atmospheric meteorological data from 26 July 2019 to 26 July 2020 were taken as samples, in which the trend term $T_{a}$ of atmospheric meteorology was x, and the micrometeorological $T_{\mu }$ was y. The correlation equation of the various meteorological data was as follows:

Relative humidity: $T_{\mu ,i}(RH)=30.35+0.6565T_{a,i\;}\;(RH)\;i=1,2,\cdots ,n$

Wind speed: $T_{\mu ,i}\left(IWS\right)=0.4089+0.3501T_{\alpha ,i}\left(IWS\right)\;i=1,2,\cdots ,n$

Accumulated rainfall: $T_{\mu ,i}(AR)=-0.08381+0.5137T_{a,i\;}(AR)\;i=1,2,\cdots n$

$$T_{\mu ,i}\left(IWS\right)=0.4089+0.3501T_{\alpha ,i}\left(IWS\right)\;i=1,2,\cdots ,n$$

The above is the correlation between micrometeorological and meteorological data. Based on this result, if the daily change in meteorological data in the atmosphere were known, the micrometeorological data of the slope on that day could be preliminarily predicted.

### 3.5. Prediction Based on ARIMA Time Series

We selected the slope-monitoring micrometeorological temperature as an example and input it into the ARIMA prediction system. The temperature data of the monitoring module from 26 July 2019 to 30 July 2019 were downloaded from the cloud platform server before prediction. The collected data were used to establish a monitoring temperature database that could can be easily and quickly imported into the software for modeling.

**Figure 7:**
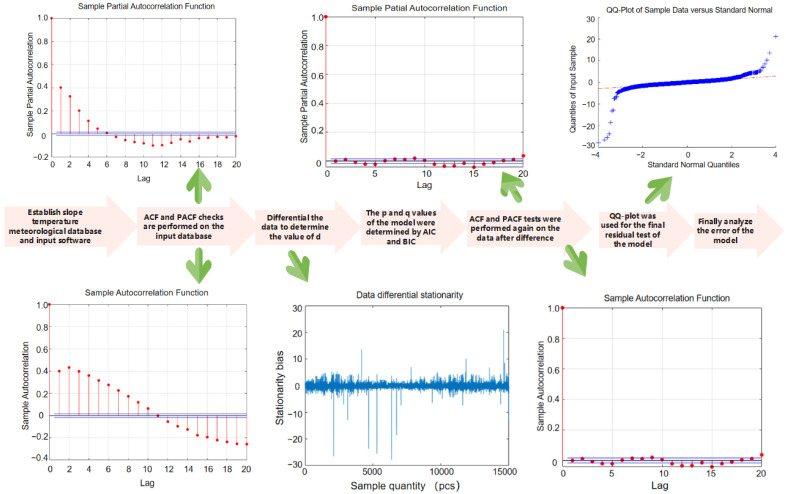
The specific process and a series of tests of establishing ARIMA time series monitoring temperature model include ACF, PACF, AIC, BIC and QQ-plot test images.

Figure 7 shows the specific process and a series of tests for establishing the ARIMA time-series monitoring temperature model. First, the stationarity test of ACF and PACF was carried out on the input monitoring temperature. The ACF image of the monitoring data showed that the automatic correction value of the sample data was too large, and the trailing did not tend to 0. Therefore, it was judged that the input monitoring temperature needed to be differentiated to ensure that the data belonged to stable time-series data, so the ARIMA model cold be effectively established.

After the first-order difference, the non-stationary trend of the model difference image was eliminated, and all the data oscillated around the mean. At this point, it could be judged that the slope monitoring temperature data had all been converted into a stable time series, and the value of d was determined to be 1. The generated model needed to test the performance of the model by explaining the relationship between variables. We used information criteria to determine how well the model interpreted the relationship. The p and q values of the model were determined by using AIC and BIC grading methods. At this time, the modeling process was completed, and the p, d and q values of the model were all determined.

To ensure that the fixed order of the model could accurately predict the future temperature trend, ACF and PACF were used for verification. It could be seen from the verified images that the automatic correction value of the sample data of the ACF and PACF images fluctuated slightly around 0 after the first-order difference, and the ACF image was extremely consistent with the PACF image. Therefore, the fixed order and difference order of the model were in line with the requirements, and there was no need to make another difference.

The QQ-plot test and Dubin–Watson residual test were the last tests conducted before model prediction. A normal distribution line was used as the main annotation position for QQ-plot test, and the sites with low front-end significance in the figure kept approaching the normal distribution line. Most of the data values in the middle were close to the straight line, and the linearity of the residuals of the quantiles followed a linear relationship. We think this is a reliable predictive model.

**Figure 8:**
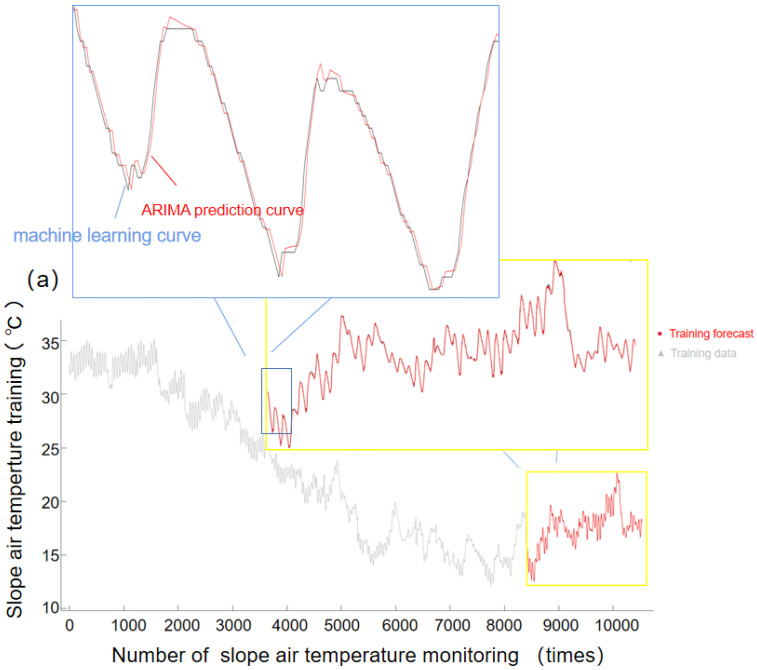

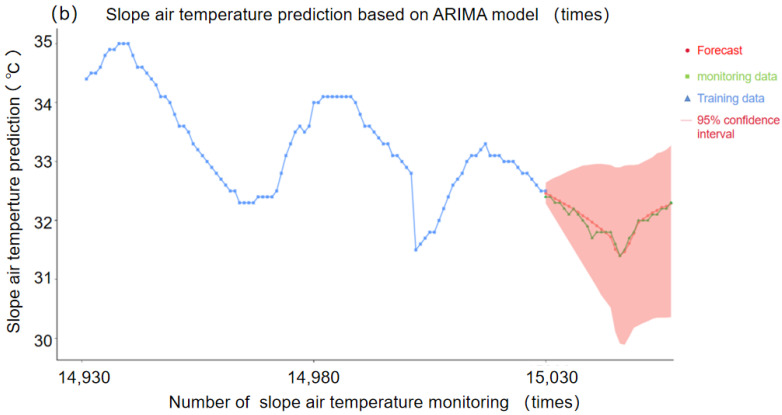
A time-series prediction model for slope air temperature monitoring based on ARIMA model, in which (**a**) training set data simulation and prediction using machine learning (**b**) comparison images of actual predicted values and monitored values.

Figure 8 shows 28 prediction images of slope temperature monitoring, in which Figure 8a is the machine learning training data, and Figure 8b is the prediction image of actual slope temperature monitoring. Through machine learning training for the first 8500 groups of data and simulation prediction, it could be seen that the prediction curve of machine learning almost completely fit the actual slope monitoring data, and the accuracy of the prediction was greatly improved through the machine learning of a large amount of data. Figure 8b shows the comparison images of actual predicted values and monitored values, in which the blue line represents the machine learning of predicted data; the red line represents the prediction data of the ARIMA model built by machine; the green line represents the monitoring data of slope; and the pink area represents the 95% confidence interval. As can be seen from the figure, the predicted data were basically consistent with the actual monitoring data, which predicted the rising and falling temperature trend. Though the prediction data can roughly get the temperature change trend and change size, it can effectively advance the protection of slope planting vegetation to a certain extent, and prevent extreme weather conditions from damaging vegetation, which can affect slope stability.

## 4. Discussion

### 4.1. Meteorological Data Analysis and Siscussion

According to Figure 4 and Figure 5, the comparison between the slope micrometeorological monitoring data and the CMDSC shows that meteorological data and the slope micrometeorological monitoring data had the same change trend, which verifies the completeness and good quality of the slope micrometeorological monitoring module.

From the comparison of daily accumulated rainfall and instantaneous wind-speed monitoring, the fluctuation of the monitoring data from the CMDSC was significantly higher than the slope micrometeorological. The daily average temperature and relative humidity measured from the CMDSC were obviously lower than those measured at for the slope micrometeorological data. This is because for rainfall and instantaneous wind speed, slope micrometeorological sensor monitoring was limited to the influence of the nearby environment. There is a forest ecosystem near the selected experimental site, and a large number of trees can slow down the wind speed and intercept rainfall to a certain extent, resulting in significantly larger values measured by the slope micrometeorological sensor. The relative humidity and average temperature of the slope were obviously higher than that of the atmosphere because of the slope soil and forest. The forest has high relative humidity, and in the daytime, the open-space drop rate of the relative humidity and average temperature is significantly higher than that of the forest environment where the sensor is located. Water and heat are difficult to volatilize, and a small part of them is deposited near the slope, resulting in higher relative humidity and average temperature monitored by the slope micro-meteorological sensor than those monitored by the atmospheric weather station.

### 4.2. Error Analysis of Micrometeorological Fitting System

Table 1 shows the record of fitting parameters of micrometeorological slope temperature and atmospheric temperature data by a first-order Fourier function. According to the discussion and analysis of the micrometeorological and atmospheric data-fitting system, we know from meteorology that the variation cycle of atmospheric temperature is 1 tropic year—365 or 366 days. Since the slope micrometeorological and atmospheric temperature change rules are basically the same, the uniform variation cycle of slope micrometeorological temperature is T = 365 days. Sampling was taken 48 times every day, so the circle frequency ω = 0.000359 = 2π*/*(365 × 48), while the circle frequency ω of the actual fitting was 0.0003868. This was due to the maintenance and inspection of the sensor equipment, which meant that the sensor did not normally monitor the micrometeorological data for several days; therefore, the actual circle frequency was high. We used the actual fitted circular frequency as the standard value.

Table 2 shows the recorded Fourier function fitting and fitting errors between the two sets of data. The sum of squares error (SSE), determination coefficient (R-Square) and root mean square error (RMSE) were used for error analysis. The determination coefficient represented the quality of the fitting through the change of data. The closer it was to 1, the stronger the variable of the equation for explaining y, and the better the model fit the data. The R-square fitting degree of atmospheric data and slope monitoring data was 85% and over 95%, which was a very good fit for large volume data. From 00:00 on 26 July 2019, atmospheric temperature data was monitored every four hours and slope data every half hour, so the amount of atmospheric temperature data was lower than slope temperature monitoring data. As a result, the SSE for the slope monitoring data was significantly greater than for the atmospheric data, but the slope monitoring data’s R-square and RMSE were much better than those of the atmospheric data. This was because the slope monitoring data of the meteorological measurement time interval was short for such a large amount of data, so it was obvious that the trend term of the Fourier function fit better.

### 4.3. Error Analysis of ARIMA Prediction System

We present the forecast data and monitoring data in Table 3, and the MAE, MAPE, MSE, RMSE are shown in Table 4. In this way, the accuracy and merits of the model can be judged intuitively.

It can be seen from Table 4 that the values of the MSE, MAE, RMSE and MAPE of the slope micrometeorological temperature monitoring data were very small, which indicated that the slope temperature monitoring data was in good agreement with the predicted value of ARIMA model with small deviation. This was mainly due to machine-learning training and the difference of a large amount of data, so the ARIMA model had a good optimization effect. Such good prediction results can be effectively combined with the fitting system. The long-term monitoring and prediction of slope weather station data were completed with data from the national weather station.

## 5. Conclusions

The slope micrometeorology is usually affected by various environmental factors, resulting in a partial difference from national atmospheric data. Therefore we developed a new slope micrometeorological monitoring system. The SMMPS innovates and upgrades the cloud platform with the addition of an ARIMA prediction system and a data-fitting system. The whole system can obtain the slope meteorological monitoring data through the slope micro-meteorological monitoring module in the early stage and finally use the data-fitting and ARIMA prediction systems to gain advanced warning of danger to the slope micro-climate, such as from high temperature and low temperature, to protect the growth of slope vegetation.

Applying the SMMPS to engineering projects, the following conclusions were obtained through an analysis of the results:

(1) In the early stage, the SMMPS can log into the cloud platform server through the remote computer client to obtain data that had been automatically monitored and uploaded by sensors, which do not need to read the data on site, thereby reducing labor costs.
(2) There was a strong correlation between slope micrometeorological and atmospheric data, but the fluctuation of some slope micrometeorological factors were much lower than those of the atmospheric data due to various environmental factors.
(3) The meteorological fitting system of the SMMPS can establish the relationship between atmospheric meteorological and slope micrometeorological data, so that the slope does not need long-term sensor monitoring. The system can effectively reduce the labor and instrument costs of long-term sensor monitoring, and only need CMDSC data to be input to get the relevant slope micrometeorological data.
(4) The ARIMA prediction module of the SMMPS can accurately predict future slope meteorological data. It can effectively protect the slope from the advent of harsh conditions, such as high temperature and low temperatures, which result in further slope instability or even damage, causing engineering construction delay.

In the following research, we found many areas to be improved, including continuously optimizing the system architecture of the cloud platform server, improving the accuracy of the data-fitting and ARIMA prediction systems, and providing more methods for the effective prediction of uncommon events. This requires us to continue to upgrade and innovate the cloud platform to ensure that the data-fitting system continuously improves the filtering of the uploaded data. To improve prediction accuracy, we also need to import atmospheric data into the data-fitting system in real time. When the difference between slope microclimate and atmospheric climate is too large, the automatic feedback sensor will re-monitor the microclimate, which can greatly reduce interference caused by systematic errors.

The whole system has been fully applied and run in some projects and has achieved success and recognition. We also hope to apply the SMMPS to more projects to achieve full automation and low-cost monitoring.

## Figures and Tables

**Table 1:** First-order Fourier function records fitting parameters of micrometeorological slope temperature data and atmospheric temperature data.

First-Order Fourier Fitting Parameters	a	b	c	𝜔 (Calculate)	$T_{m}$	$\Delta T_{R}$	φ
Micrometeorological slope temperature data	24.27 (24.23, 24.32)	9.165 (9.134, 9.196)	−1.224 (−1.348, −1.1)	0.000359	24.27	9.246	−82.39
Atmospheric temperature data	17.93 (17.42, 18.44)	10.26 (9.712, 10.81)	1.487 (−0.0174, 0.0190)	0.01823	17.93	10.367	81.75

**Table 2:** Fourier function is used to fit the fitting error between micrometeorological slope temperature data and atmospheric temperature data.

First Order Fourier Fitting Error	SSE	R-Square	RMSE	ω (Actual)
Micrometeorological slope data	3.297e+04	0.9549	1.423	0.0003868
Air data	3150	0.8681	2.946	0.01823

**Table 3:** ARIMA time series model is used to compare the prediction of slope micrometeorological monitoring temperature.

Frequency of Slope Temperature Monitoring	Actual Temperature Monitoring Value (°C)	ARIMA Temperature Prediction Value (°C)	95% Confidence Interval Maximum Predicted Value (°C)	95% Confidence Interval Minimum Predicted Value (°C)
15,030	32.4	32.46	32.29	32.64
15,031	32.4	32.42	32.16	32.69
15,032	32.3	32.37	32.03	32.73
15,033	32.3	32.33	31.90	32.76
15,034	32.2	32.28	31.78	32.80
……	……	……	……	……
15,052	32	32.08	30.29	33.03
15,053	32.1	32.13	30.33	33.07
15,054	32.1	32.17	30.35	33.14
15,055	32.2	32.22	30.35	33.16
15,056	32.2	32.24	30.35	33.20
15,057	32.3	32.29	30.36	33.27

**Table 4:** ARIMA time series model is used to compare the prediction of slope micrometeorological monitoring temperature.

Slope Micrometeorological to Predict	ARIMA (p,d,q)	MSE	MAE	RMSE	MAPE	D–W
Prediction of Slope temperature	(7,1,7)	0.00671	0.0611	0.082	0.00191	2.0001

## Data Availability

The data presented in this study are available on request from the corresponding author.
